# Estimated Prevalence and Care Pathway of Feeding and Eating Disorders in a French Pediatric Population

**DOI:** 10.3390/nu13062048

**Published:** 2021-06-15

**Authors:** Valérie Bertrand, Lyvia Tiburce, Thibaut Sabatier, Damien Dufour, Pierre Déchelotte, Marie-Pierre Tavolacci

**Affiliations:** 1 Pediatric Unit, Le Havre Hospital, 76083 Le Havre, France; tiburce.lyvia@gmail.com; 2 French National Institute of Health and Medical Research (INSERM) U1073, UNIROUEN, Normandie University, 76031 Rouen, France; Pierre.Dechelotte@chu-rouen.fr (P.D.); mp.tavolacci@chu-rouen.fr (M.-P.T.); 3 Clinical Investigation Center 1404, Rouen University Hospital, 76031 Rouen, France; thibaut.sabatier1@gmail.com; 4 Pediatric Emergency Care, Le Havre Hospital, 76083 Le Havre, France; damien.dufour@ch-havre.fr; 5 Department of Nutrition, Rouen University Hospital, 76031 Rouen, France

**Keywords:** feeding and eating disorders, pediatrics, prevalence

## Abstract

Feeding and Eating Disorders (FED) are mostly described in infants and adolescents but are less well-known in children. Information on the prevalence of FED in the general pediatric population is still limited. The aim of this study was to estimate the prevalence and the care pathway of FED in a population aged 0–18 years old, using the Diagnostic and Statistical Manual of Mental Disorders (DSM)-5 classification. Two physicians interviewed 401 families using a questionnaire including demographics, BMI, dietary behavior data, and age-appropriate screening tools. Qualitative and quantitative variables were compared using the Chi^2^ test and Student’s t-test, respectively. After a headcount adjustment based on the French population by age group, the estimated prevalence rate was 3% [95%CI (1.7–5.1)] for Avoidant and Restrictive Food Intake Disorder (ARFID), and 9.7% [95%CI (7.2–13.0)] for Unspecified FED (UFED), which included other restrictive and compulsive FED. The median age for ARFID was 4.8 years (0.8–9 years), and 7.5 years (0.6–17 years) for UFED. The interviews did not identify cases of anorexia, bulimia, binge eating disorder, other specified FED, pica or rumination. Only 15.2% of children with an FED were receiving medical care. The development of validated pediatric screening tools, as well as the training of health professionals in children FED is necessary.

## 1. Introduction

The knowledge of Feeding and Eating Disorders (FED) in children and adolescents has evolved significantly since the 2000s. Early descriptions of pediatric eating disorders (ED) were limited in the DSM-III (Diagnostic and Statistical Manual of Mental Disorders) to pica or rumination, while anorexia nervosa (AN) and bulimia nervosa (BN) were described in adolescents in the same way as in adults [1]. Later studies have contributed to a better understanding of FED in young children [2,3]. Accordingly, the DSM-IV included for the first time in 1998 a chapter dedicated to ED in children aged up to 6 years old [1], but many children were identified as having “ED not otherwise specified”. Subsequently, the classification of GOSH (Great Ormond Street Hospital, London, UK) was proposed for children aged from 6 to 13 years old, which included early AN, restrictive eating, selectivity/neophobia, emotional food avoidance, functional dysphagia and food refusal [4,5]. The first cases of binge eating disorders (BED) in children were also described in the 2000s [6,7]. In order to include children over 6 years old [8], the new DSM-5 classification (2013) suppressed the age limit and proposed a classification based on types of FED [1]. This included new diagnostic categories, notably Avoidant and Restrictive Food Intake Disorder (ARFID), BED, Other Specified FED (OSFED) and Unspecified FED (UFED). In contrast to AN and BN, ARFID is not associated with weight and shape concerns. ARFID includes restrictive eating, selective eating, phobia of swallowing and/or vomiting and food avoidance emotional disorder. The definition states that ARFID is an apparent lack of interest in eating or food and/or an avoidance based on the sensory characteristics of food and/or concern about aversive consequences of eating, associated with one (or more) of the following: significant weight loss, significant nutritional deficiency, dependance on enteral feeding or oral nutritional supplements, marked interference with psychosocial functioning. When ARFID is associated with a concurrent medical condition or mental disorder, its severity exceeds the eating disturbance routinely associated with these troubles, which makes classification difficult to follow in some practical cases [9]. OSFED include atypical AN, BN, BED, purging disorder, night eating syndrome and UFED associate clinically significant distress or impairment in social, occupational, or other important areas of functioning predominate but do not meet the full criteria for any of the disorders in the FED diagnostic class. 

Although interest in FED has increased with DSM-5, prevalence studies in the general pediatric population are still limited, particularly in pre-pubertal children [10]. Pediatricians are actually aware of feeding disorders (FD) in infants, and of ED (AN and BN) in adolescents. In contrast, ED in toddlers (1–3 years old), and ED in the first and middle childhood (3–12 years old), are less well known. The aim of the present study was to estimate the prevalence of FED in a population of children and adolescents aged from 0 to 18 years old, using the DSM-5 classification. Secondary objectives were to correlate FED diagnosis with BMI, and to assess the care pathway. Our hypothesis was that patients with FED were poorly cared for, particularly in children aged from 1 to 12 years old. 

## 2. Materials and Methods

### 2.1. Study Design 

We carried out a non-interventional cross-sectional study in one French department (Seine-Maritime, Normandy), from 3 May 2019 to 5 March 2020. The protocol was approved by a Committee for the Protection of Persons on 18/03/2019 (N° RCB 2019-A00067-50). During general pediatric consultations, two pediatricians invited families to participate in the study with an information sheet. These consultations were conducted in a pediatric department. Patients were seen consecutively in order of arrival, without selection, until reaching a total of 400 patients, almost 100 patients in each of four age groups (0–1 year old, 1–6 years old, 6–12 years old and 12–18 years old). Physicians used an anonymized questionnaire to conduct the interview, which included demographic, medical, and age-appropriate dietary behavior data. Parents and adolescents also completed a part of the questionnaire with age-appropriate screening tools. The estimated participation time was 20 minutes. Physicians helped families if questions were not understood.

### 2.2. Inclusion Criteria

Children aged from 0 to 18 years were included, regardless of the reason for consulting and of their previous medical condition. Exclusion criteria were patients who were over 18 years of age on the day of the interview, parents or adolescents who were illiterate or unable to answer the questionnaire, patients who were not in a good physical or mental state to answer the questionnaire at that time or who did not agree to participate.

### 2.3. Data Collected in the Questionnaires

#### 2.3.1. Demographic and Medical Data

Each questionnaire included, for all patients, demographic data (age, gender), medical data (physicians measured weight, height, body mass index (BMI), listed reason for consultation, personal history), and questions targeting parents’ feelings about their child’s current eating disorder (perception of the child’s eating difficulties, which care pathway). Overweight and underweight were assessed according to the latest validated French growth curves [11]. After 2 years of age, children were considered underweight if BMI ≤ International Obesity Task Force (IOTF) 17 (grade II) and overweight if BMI ≥ IOTF 25.

#### 2.3.2. Age-Appropriate Dietary Behavior Questions and Additional Screening Tools

Questionnaires also included age-appropriate dietary behavior questions and additional screening tools. These tools were extracted from the literature according to feasibility criteria, including completion time:

- For infants aged less than 1 year old, if the infant was fed only with milk, the type of feeding was specified (breast and/or bottle feeding), and parents completed the Baby Eating Behavior Questionnaire (BEBQ), which explores the enjoyment of food, food responsiveness, slowness in eating, satiety responsiveness and general appetite [12]. If the infant had started food, parents were asked about dietary diversification and then completed the Behavioral Pediatrics Feeding Assessment Scale (BPFAS), which evaluates the child’s behavior and the parents’ feelings about or strategies for dealing with eating problems, giving three scores (child, parent, total frequency score) [13,14]. BEBQ and BPFAS do not have a validated cut-off.
- For children aged from 1 to 12 years old, parents were asked about the child’s eating behavior and then completed the BPFAS questionnaire.
- For adolescents, parents were asked about their eating behavior, and adolescents completed three screening questionnaires: the Sick Control One Fat Food, French (SCOFF-F), which is positive when the score is ≥2 [15,16], the Eating Attitudes Test EAT-26, which is positive when the score is ≥20, and explores dieting, bulimia and oral control [17,18], they are both predictive for eating disorders, and the Three-Factor Eating Questionnaire (TFEQ-R18), which does not have a cut-off, but evaluates cognitive restriction, uncontrolled eating and emotional eating [19,20].

### 2.4. Classification of Disorders According to DSM-5

After each interview, the physicians allocated one or more eating behavior characteristic to each patient (“FED items”), according to a predefined grid and in blind-fashion regarding screening tool scores, which were calculated later: no FED, breastfeeding difficulties, difficulty taking the bottle, refusal of the spoon, refusal of pieces, big eater, picky eater, neophobic/selective, restrictive with nutritional or psycho-social consequences, phobia of swallowing and/or vomiting, emotional food avoidance, hyperphagia, tachyphagia, nibbling between meals, compulsive eating, nocturnal nibbling, nocturnal hyperphagia, rumination, pica, AN, BN, BED, orthorexia. Then each patient with FED items was classified according to the DSM-5 classification (AN, BN, BED, ARFID, OSFED, pica, rumination, or UFED). Only significant and problematic isolated or associated FED items were used to classify patients’ FED. For example, isolated items like tachyphagia or nibbling were not considered.

### 2.5. Statistical Analysis

A descriptive analysis was done for the entire cohort and for each age group. The analysis included sex ratio, age, BMI, FED prevalence rates, screening tool scores and the care pathway. Qualitative variables were described in percentages, and quantitative variables were described with medians (minimal-maximal) for age, or with means for score results (±standard deviation SD). When data on some items were missing in screening tool questionnaires, raw scale scores were transformed to a 0–100 scale [(raw score − lowest possible raw score)/possible raw score range)/100]. A headcount adjustment based on the French population by age group [21] was carried out for the overall calculation of FED prevalence from 0 to 18 years old. Statistical analysis was done with the software XlstatBiomed 2020.3.1 (Addinsoft, Paris, France). The normality of the distribution was checked with the Shapiro-Wilk test (*p* > 0.05). Chi-square was used for comparisons of categorical data. Continuous variables were described with means, SD, and compared using the Student’s t-test. Ninety-five percent confidence intervals (CI) were calculated for the prevalence of FED.

## 3. Results

### 3.1. Study Sample and Demographic Data 

**Figure 1:**
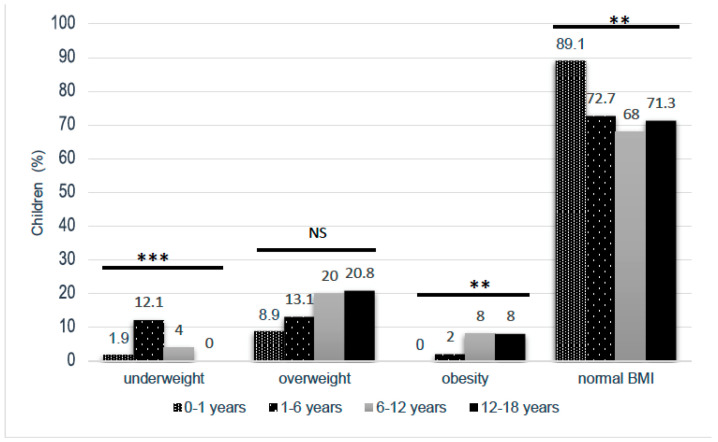
BMI data of the pediatric population, according to age group (*** *p* < 0.001, ** *p* < 0.01, NS no significant, χ^2^ test).

A total of 401 questionnaires were collected (Table 1). In the 0–1 year-old group, 58 infants were fed only with milk (only breastfeeding *n* = 20, breast and/or bottle-feeding *n* = 11, only bottle-feeding *n* = 27), and 43 older infants had started food. The reasons for consultations were very varied (medicine 77.3%, surgery 22.0%, psychological 2.7%). Only three consultations were nutrition-related (one follow-up for obesity and two infants for decreased appetite, without a known FED). The prevalence of overweight (including obesity) was 20.2% (markedly higher over the age of 6 years), and the prevalence of underweight was 4.5% (most prevalent in the 1–6 years age group) (Figure 1).

### 3.2. FED Items

A total of 427 FED items were identified. The percentage of eating behavior characteristics was calculated according to the age groups concerned (Table 2). Since some items were age-specific, we could not compare the global prevalence of items across age groups; however, the highest number of items was observed in the 12–18 years age group. There were more restrictive FED items in the 1–6 years age group (picky eater, restrictive eater), and more compulsive FED items in adolescents (hyperphagia, nocturnal nibbling, tachyphagia, nibbling between meals). The item “selective eater” was found in all age groups with a frequency of 2–9%. Eighteen percent of selective eaters had severe selectivity (less than 10 different foods): they were aged between 3 and 4.5 years old, and one of them had an autism spectrum disorder.

### 3.3. Prevalence of FED According to DSM-5

In a second step, we classified patients having FED according to the DSM-5 classification and the estimated prevalence was calculated for each age group (Table 3). We identified 2.7% of patients with ARFID, median age 4.8 years (0.8–9 years) and 8.7% of patients with UFED, median age 7.5 years (0.6–17 years), *p* = 0.09. Children who had a marked selective eating behavior, but without other ARFID diagnostic criteria, were classified as UFED. The interviews did not identify any other FED characterized by DSM-5, such as AN, BN, BED, OSFED, pica or rumination. After a headcount adjustment based on the French population by age group, the total prevalence of FED estimated from this cohort was 12.7% [95%CI (9.8–16.3)]. The prevalence of ARFID was 3.0% [95%CI (1.7–5.1)] and the prevalence of UFED was 9.7% [95%CI (7.2–13.0)]. 

### 3.4. FED and BMI 

Patients with ARFID were more often underweight (91%) than patients with UFED (0%) and patients without FED (2.2%) (*p* < 0.001). Children aged between 1 and 18 years who had tachyphagia were more often overweight (40%) than children without tachyphagia (16%) (*p* < 0.001). Patients with nibbling were more often overweight (34.8%) than those without (19.4%) (*p* = 0.004). Compulsive eaters were more often overweight (80%) than non-compulsive eaters (23%) (*p* = 0.001). Among the 29 overweight adolescents, 13.8% were compulsive eaters. 

### 3.5. FED and Analysis of Screening Tools 

**Figure 2:**
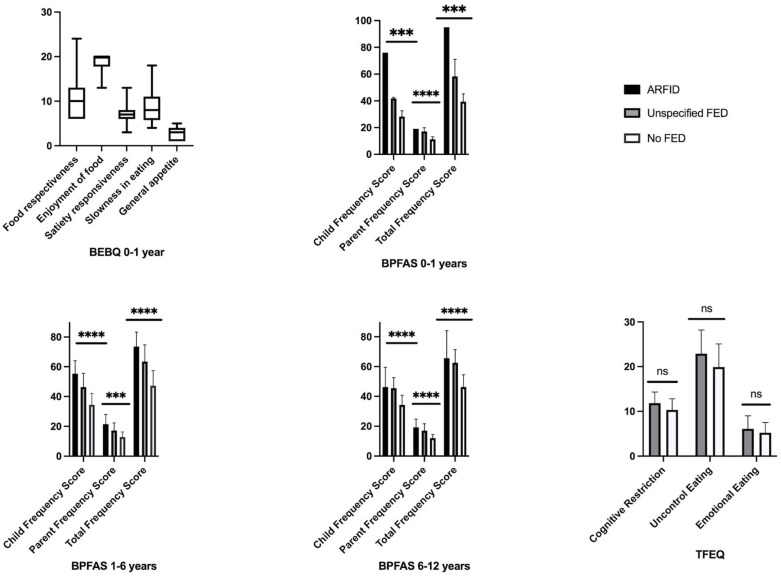
Means of BEBQ (baby eating behavior questionnaire), BPFAS (Behavioral Pediatrics Feeding Assessment Scale) and TFEQ-R18 (Three-Factor Eating Questionnaire) scores (±standard deviation) according to the age and DSM-5 classification of the patients (*** *p* < 0.001, **** *p* < 0.0001, Student’s t-test).

The means of BEBQ, BPFAS and TFEQ scores according to the DSM-5 classification of patients are presented in Figure 2. Parents answered all questions except for the BPFAS in infants, where a few responses were missing. For infants who had not started food, BEBQ scores were consistent with the parents’ interview (no FED in this group). For all other children, patients with ARFID had the highest BPFAS scores. For adolescents, 23 SCOFF-F and 2 EAT-26 scores were positive. Among positive SCOFF-F, 3 patients had an UFED, and 20 patients had no FED diagnosis, but only one of them also had a positive EAT-26 score.

### 3.6. FED Care Pathway 

Among the cohort, 20% of parents felt their child had feeding or eating difficulties. More difficulties were found with adolescents than with other age groups. Overall, 79.7% of families felt this eating difficulty was problematic or alarming, but only 22.3% of children were receiving professional care, mostly infants, with the 1–6 and 6–12 years-old groups receiving the least care (Table 4). When considering only the FED classified according to the DSM-5, 87.8% of parents felt it was problematic or alarming (ARFID 88.9%, UFED 87.5%), but only 15.2% of children were receiving professional care (ARFID 18.0%, UFED 14.2%), with the least care in the 1–6 and 6–12 years-old groups (20% and 11% respectively), in contrast to the infants group (50%) and the adolescents group (25%).

## 4. Discussion

In this large cohort, FED were diagnosed in at least 12.7% of the children and adolescents, including 3% with ARFID. Few studies have assessed the prevalence of FED in the general pediatric population [22]. Most epidemiological studies included adults, using the DSM-IV classification, or the DSM-5 posteriori on previous DSM-IV data, including sometimes adolescents [23,24,25]. The main limiting factor in assessing pediatric FED prevalence is probably the lack of tools that can screen all FED, regardless of age. 

### 4.1. Prevalence of ARFID and Related Subtypes

ARFID is described from the age of 1 year until adolescence, and patients with ARFID are generally younger than those with AN or BN [26,27,28,29,30,31]. For infants, ARFID should not be confused with a normal and transient neophobia at the age of 6–9 months, or with a marked selective eating behavior that would not include the associate clinical features described in the ARFID DSM-5 definition and could be then classified as UFED.

Two studies screened ARFID in school children. ARFID was identified in 3.2% of 1444 Swiss children aged from 8 to 13 years [32], using the Eating Disturbances in Youth-Questionnaire (EDY-Q). In 4816 Taiwanese children aged from 7 to 14 years old, ARFID prevalence was estimated at 0.5%, using a mental-disorder questionnaire with psychiatric interviews [33]. For the same age group, ARFID prevalence was 2.8% in our study. Percentages of sub-types in our study were 63% (restrictive), 54.5% (selective), and 10% (phobia eaters), thus rather similar to 39%, 60.9% and 15.2 %, respectively, in the Swiss study [32].

Three studies have investigated the 8–18 years age group. A retrospective study in a gastroenterological pediatric cohort [28] identified 1.5% ARFID in 2250 children, (57.6% restrictive, 21% selective, 9% phobias eaters). The EDY-Q, without clinical interview, detected 3.7% ARFID in 190 girls followed in a pediatric gynecology clinic [34] and 0.9% ARFID in 111 children from a pediatric hospital, as compared to 2.4% in a general population sample [35]. Thus, our estimated prevalence of 3% of ARFID fits well in the range of other European studies. 

For toddlers, data on the prevalence of ARFID are lacking. Before the diffusion of DMS-5, a study in 400 children aged 1–4 years old identified 15.4% selective, 11.2% restrictive, and 0.25% phobia eaters [36], versus 5.9%, 4.7% and 3.5% respectively, in our study for the same age group. The “selective” and “restrictive” profiles are often called “picky eater” in the literature and are most frequent between 2 and 6 years old [36,37,38]. Recently, a retrospective study on insurance databases of children up to 5 years in the USA reported FD in 21–34 children for 1000 child-years [39], which underlines the need for active screening of FD [40]. 

### 4.2. FED Other Than ARFID

In our study, we did not identify AN, BN, BED or OSFED. As a matter of fact, their prevalence is generally low, but we cannot exclude that some adolescents had debutant FED not revealed during the interview. AN prevalence is estimated at approximately 0.3–0.9% in adolescents and 0.2% in children [10,24,33]. The prevalence of BN, BED and pica/rumination is not well-known and is estimated at 0.3% [41,42], 1–3% [6,43], and 1.5–10% [10,44,45], respectively. BN and BED are more frequent in adolescents and adults [46]. In overweight children/adolescents, one metanalysis reported 22% of compulsive disorders [47]. Night Eating Disorders are not well documented [48,49]. In pediatric FED series, AN remains the most common FED (40–70%), followed by ARFID (5–22.5%), and BN (4–12%) [27,29,30,31,50,51,52]. It is probably because AN results generally in a lower BMI than ARFID and is thus more likely to be identified and treated than ARFID [27,30,32,33,53]. 

A majority of children with FED in our cohort were classified as UFED. Indeed, the DSM-5 classification remains criticized because definitions of specified FED are not always usable in children [9,43]. Pediatric FEDs have multifactorial origins (aversive environmental factors, association with organic and/or mental diseases), resulting in complex clinical features with varied levels of severity [27,54]. It is estimated that 25–45% of young children in the general population experience FD at some point, 5–10% requiring intensive management [8,55,56]. Recently, some authors have therefore proposed a functional or a severity approach of FD in four areas (medical, nutritional, oral developmental and psychosocial) [57,58]. It is important to better identify children with UFED, because although it is less severe than other disorders, they may require parental guidance in order to prevent the disorder from worsening to full ARFID or even ED [55,59,60]. 

### 4.3. Insufficient Care of ARFID and UFED

In our study, only 15.2% of children with identified ARFID and UFED were already receiving care. These FED are probably not well-known by physicians and are difficult to diagnose [61]. Delays in ARFID diagnosis of up to 33 months, much longer than for AN, have been reported [27,30]. In our experience, BPFAS is helpful in detecting restrictive, selective eating in children between 10 months and 10 years old, but not specifically ARFID. In children after 10 years, SCOFF or EAT26 are helpful tools to screen for AN and BN, while TFEQ is helpful to screen for compulsive ED. Although SCOFF has also been validated in adolescents, we observed in our study that some questions of the SCOFF-F were not always easy to understand for some adolescents [15]. Several tools have been proposed to screen for ARFID in children, but none is validated for all age groups thus far [32,62,63]. Therefore, only structured interviews allow for the diagnosis of FED. 

### 4.4. Strength and Limitations of the Study 

Our study is one of the rare pediatric studies based on clinical interviews to estimate prevalence rates and the care pathway of FED in a general population. Our population was quite representative of the French population in terms of sex ratio and BMI [64]. Our observed high prevalence of overweight in children aged between 6 and 18 years old is consistent with that observed in the Normandy region of France and European countries [65,66]. Our population size was sufficient to detect most FED represented by UFED and ARFID, but too small to give an estimate of the less prevalent AN, BN or BED in pediatrics. Interviews alone may also have limitations, because some differential diagnoses of FED may remain unidentified. In addition, some adolescents could under-declare their symptoms, and parents may also misperceive their child’s eating behavior [36,55,67]. 

## 5. Conclusions

Although ARFID and UFED are present in 12.7% of pediatric patients, we can conclude that they remain largely underdiagnosed because the care pathway is not correlated with the parents’ request for care. We suppose that the clinical features of ARFID and UFED are not well known and are difficult to identify by untrained physicians, leading to delayed care. Moreover, this study shows that the DSM-5 classification remains poorly adapted to children, because the most frequent disorders (UFED) are the least well defined. UFED requires further clinical studies to better characterize less severe atypical subtypes of FED, which are common in children. The development of validated screening tools, as well as the training of health professionals in all clinical forms of pediatric FED are necessary. Early screening for pediatric FED should be performed to prevent both nutritional and psychopathological consequences.

## Figures and Tables

**Table 1:** Demographic data and BMI data of the pediatric population, by age group.

	Age Groups (Total *n* = 401 Patients)				
	0–1 Year Old *n* = 101	1–6 Years Old *n* = 99	6–12 Years Old *n* = 100	12–18 Years Old *n* = 101	*p* *
Sex ratio	1.2	0.86	1.2	1.1	0.58
Median age (min-max)	3.5 months (0.1–11.9)	3 years old (1–5.5)	8 years old (6–11.5)	14 years old (12–18)	
BMI overweight (including obesity)	8.9%	15.1%	28%	28.7%	<0.001
BMI grade II underweight	1.9%	12.1%	4%	0%	<0.001

* χ^2^ test was used for comparisons of categorical data, Student’s t-test was used for comparisons of continuous variables.

**Table 2:** Prevalence of feeding and eating disorders (FED) items by age group.

	Age Groups (Total *n* = 401 Patients, 427 FED Items)				
*n* = FED Item/Patient (%)	0–1 Year Old *n* = 101 Infants *n* = 23 Items	1–6 Years Old *n* = 99 Children *n* = 112 Items	6–12 Years Old *n* = 100 Children *n* = 133 Items	12–18 Years Old *n* = 101 Adolescents *n* = 159 Items	*p* (χ^2^ Test)
Breastfeeding withdrawal problems *n* = 2/31(6.4%)	2/31 breast-feeding (6.4%)	0/99 (0%)	NA	NA	NA
Difficulty taking the bottle *n* = 0 (0%)	0/101 (0%)	0/99 (0%)	NA	NA	NA
Refusal of the spoon *n* = 2/142 (1.4%)	2/43 infants who had started food (4.6%)	0/99 (0%)	NA	NA	NA
Refusal of pieces *n* = 10/343 (2.9%)	4/43 infants who had started food (9.3%)	6/99 (6%)	0/100 (0%)	0/101 (0%)	0.004
Picky eater *n* = 66/401 (16.4%)	6/101 (6%)	30/99 (30%)	17/100 (17%)	13/101 (13%)	<0.001
Neophobic/selective eater *n* = 22/343 (6.4%)	1/43 infants who had started food (2.3%)	7/99 (7%)	9/100 (9%)	5/101 (5%)	0.57
Restrictive eater *n* = 7/343 (2%)	0/101 (0%)	6/99 (6%)	1/100 (1%)	0/101 (0%)	0.01
Phobia of swallowing and/or vomiting *n* = 4/300 (1.3%)	NA	3/99 (3%)	1/100 (1%)	0/101 (0%)	0.16
Emotional food avoidance *n* = 1/300 (0.3%)	NA	0/99 (0%)	1/100 (1%)	0/101 (0%)	NA
Big eater *n* = 93/401 (23.2%)	8/101 (8%)	25/99 (25%)	29/100 (29%)	31/101 (31%)	0.001
Hyperphagia *n* = 20/343 (5.8%)	NA	0/99 (0%)	8/100 (8%)	12/101 (12%)	0.003
Compulsive eating *n* = 5/343 (1.4%)	NA	0/99 (0%)	1/100 (1%)	4/101 (4%)	0.07
Nocturnal nibbling *n* = 4/343 (1.1%)	NA	0/99 (0%)	0/100 (0%)	4/101 (4%)	0.02
Nocturnal hyperphagia *n* = 1/343 (0.3%)	NA	0/99 (0%)	0/100 (0%)	1/101 (1%)	0.37
Tachyphagia *n* = 101/343 (29.4%)	NA	21/99 (21%)	30/100 (30%)	50/101 (50%)	<0.001
Nibbling between meals *n* = 89/343 (25.9%)	NA	14/99 (14%)	36/100 (36%)	39/101 (39%)	<0.001
Anorexia or bulimia nervosa, binge eating disorder, rumination, pica, orthorexia *n* = 0/300	NA	0/99 (0%)	0/100 (0%)	0/101 (0%)	NA

Abbreviations: FED feeding and eating disorders, NA not applicable.

**Table 3:** Prevalence of patients with feeding and eating disorders (FED) according to the DSM-5 classification.

	Age Groups (Total *n* = 401 Patients)				
DSM-5 FED *n* = 46/401 (11.4%)	0–1 Year Old *n* = 101	1–6 Years Old *n* = 99	6–12 Years Old *n* = 100	12–18 Years Old *n* = 101	*p* (χ^2^ Test)
ARFID *n* = 11/401 (2.7%)	*n* = 1 (1%)	*n* = 6 (6%)	*n* = 4 (4%)	n = 0	
Selective *n* = 3 (including 1 refusal of pieces)	1	0	2	0	0.09
Emotional food avoidance *n* = 1	0	0	1	0	
Restrictive eater *n* = 7 (including 1phobia SV, 3 selective eaters)	0	6	1	0	
Unspecified FED *n* = 35/401 (8.7%)	*n* = 6 (6%)	*n* = 8 (8%)	*n* = 9 (9%)	*n* = 12 (12%)	
Compulsive eating *n* = 4	0	0	1	3	
Nocturnal snacking/hyperphagia *n* = 5 (including 1 selective)	0	0	0	5	
Refusal of pieces + phobia SV *n* = 1	0	1	0	0	
Refusal of pieces + selective *n* = 2	0	2	0	0	
Selective eater *n* = 12	0	1	7	4	
Phobia SV + selective eater *n* = 1	0	1	0	0	
Phobia SV *n* = 1	0	0	1	0	
Refusal of pieces *n* = 6	3	3	0	0	
Refusal of the spoon *n* = 1	1	0	0	0	
Breastfeeding withdrawal problem *n* = 1	1	0	-	-	
Refusal of the spoon + breastfeeding withdrawal problem *n* = 1	1	0	-	-	
Anorexia nervosa, bulimia nervosa, binge eating disorder, rumination, pica, other specified FED *n* = 0	0	0	0	0	

Abbreviations: ARFID avoidant and restrictive food intake disorder, DSM-5 Diagnostic and Statistical Manual of Mental Disorders-5th edition, FED feeding and eating disorders, *n* number, Phobia SV Phobia of swallowing and/or vomiting.

**Table 4:** Parents’ feelings about their child’s current eating difficulties, and the care pathway.

	Age Groups (Total *n* = 401 Patients)				
Parents’ Feelings	0–1 Year Old *n* = 101	1–6 Years Old *n* = 99	6–12 Years Old *n* = 100	12–18 Years Old *n* = 101	*p*
Having a child with current eating difficulties (20%)	11.0%	20.0%	19.0%	30.0%	0.021
Perception of the child’s eating difficulties					<0.001
It is not a problem (20.3%)	18.2%	17.6%	23.5%	20.7%	
It is a problem (52.7%)	45.4%	64.7%	52.9%	48.3%	
It is alarming (27%)	36.4%	17.6%	23.5%	31.0%	
Child receiving professional care (22.3%)	54.5%	15.0%	15.8%	26.9%	0.007
General practitioner (29.4%)	66.7%	33.3%	66.7%	-	
Pediatrician (35.2%)	33.3%	66.7%	-	28.6%	
Psychiatrist (5.9%)	-	-	-	28.6%	
Dietician (11.7%)	-	-	33.3%	42.8%	

## Data Availability

Data can be made available upon request. However, sharing of the data may require approval and some access restrictions may apply. Requests may be sent to the corresponding author.
